# Compact and modular system architecture for a nano-resonator-mass spectrometer

**DOI:** 10.3389/fchem.2023.1238674

**Published:** 2023-09-07

**Authors:** Adrien Reynaud, Wioletta Trzpil, Louis Dartiguelongue, Vaitson Çumaku, Thomas Fortin, Marc Sansa, Sebastien Hentz, Christophe Masselon

**Affiliations:** ^1^ Université Grenoble Alpes, CEA-Leti, Grenoble, France; ^2^ Université Grenoble Alpes, CEA, Institut de Recherche Interdisciplinaire de Grenoble, Grenoble, France; ^3^ INSERM UA13 Biosciences et bioingénérie pour la santé, Grenoble, France

**Keywords:** mass spectrometry, NEMS, aerodynamic lens, resonator, single particle

## Abstract

Mass measurements in the mega-to giga-Dalton range are essential for the characterization of natural and synthetic nanoparticles, but very challenging to perform using conventional mass spectrometers. Nano-electro-mechanical system (NEMS) based MS has demonstrated unique capabilities for the analysis of ultra-high mass analytes. Yet, system designs to date included constraints transferred from conventional MS instruments, such as ion guides and high vacuum requirements. Encouraged by other reports, we investigated the influence of pressure on the performances of the NEMS sensor and the aerodynamic focusing lens that equipped our first-generation instrument. We thus realized that the NEMS spectrometer could operate at significantly higher pressures than anticipated without compromising particle focusing nor mass measurement quality. Based on these observations, we designed and constructed a new NEMS-MS prototype considerably more compact than our original system, and which features an improved aerodynamic lens alignment concept, yielding superior particle focusing. We evaluated this new prototype by performing nanoparticle deposition to characterize aerodynamic focusing, and mass measurements of calibrated gold nanoparticles samples. The particle capture efficiency showed nearly two orders of magnitude improvement compared to our previous prototype, while operating at two orders of magnitude greater pressure, and without compromising mass resolution.

## 1 Introduction

The characterization of large supra-molecular species is attracting growing interest in biology and analytical chemistry (Keifer et al., 2017; Erdogan et al., 2022). Mass spectrometry (MS) in the MDa to GDa range is especially interesting for the analysis of viral particles and synthetic nanoparticles (Dominguez-Medina et al., 2018; Lai et al., 2021). In this mass range, conventional MS becomes challenged by sample heterogeneity associated with the presence of variant species, chemical modifications, as well as salt or solvent adducts (Rolland and Prell, 2022). This creates highly convoluted m/z patterns that cannot be straightforwardly converted into the original analyte’s mass. Several technologies based on single particle mass determination methods have emerged to circumvent this issue, namely charge detection MS (CDMS) (Jarrold, 2022), nano-electro-mechanical system-based mass spectrometry (NEMS-MS) (Sage et al., 2015) and mass photometry (Young et al., 2018).

Nano-electro-mechanical system-based mass spectrometry (NEMS-MS) has unique capabilities to analyze ultra-high mass analytes in the MDa to GDa mass range regardless of their charge (Hanay et al., 2012; Sage et al., 2015). Earliest NEMS-based mass spectrometers consisted of modified MS architectures in which the detector was replaced by a nano-resonator (Naik et al., 2009; Hanay et al., 2012). Employing NEMS allowed decreasing the number of components because it works as both detector and analyzer. Moreover, as NEMS-MS does not require charging of the particles, analytes focusing could also rely on particle inertia, while mass is measured directly. These features make NEMS-MS insensitive to mass to charge convolution generated by similar species with differing charge states, enabling a simplified architecture that could ultimately become integrated as tiny instruments. However, as the technique is relatively young (Naik et al., 2009), many features and components still require optimization.

The first NEMS-MS prototype (Naik et al., 2009) consisted of an electrospray ionization source (ESI), a two-stage differentially pumped hexapole ion optics driven at radio frequency, and a NEMS mass sensor localized 2 m below the source. The NEMS resonator was operated at a pressure of $10^{-8}$ mbar and cooled to 40 K. This prototype achieved the first demonstration of NEMS-based MS of single biological molecules. Subsequent research on NEMS-MS focused on simplifying the measurement and the mass spectrometer’s architecture, as well as developing more effective methods to focus the analyte on the NEMS mass analyzer/detector. A significant milestone was independently proposed by Hentz and Masselon (2016) and by Malvar et al. (2016). They proposed NEMS-MS systems devoid of ion guides, allowing them to create prototypes with somewhat relaxed pumping requirements. These systems consisted of series of chambers with decreasing pressures: a nebulization stage operating at ambient pressure, a heated capillary inlet with a first pressure drop to 10–100 mbar, a pressure-limiting orifice that could optionally be followed by a series of focusing orifices (aerodynamic lens), and a resonator chamber operating in the 10^−3^ to 10^−5^ mbar regime. These systems allowed decreasing the apparatus size, pumping requirements, and complexity of the system. However, the prototypes were still characterized by modest particle capture efficiency defined as the ratio of the number of detected over emitted particles. In the work by Malvar et al. 1 particle per $5\times 10^{8}$ was detected, with event rates of ∼0.3 particle per minute. Dominguez-Medina et al. (2018) reported the detection of 1 viral particle per $2.6\times 10^{8}$ with an event rate of 0.8–1.35 particle per minute on a 20 resonators array. Recently, Hannay’s group proposed a NEMS-MS system operating under ambient conditions while providing improved particle focusing (Erdogan et al., 2022). As a result, they achieved higher capture efficiency detecting 1 particle per $1.85\times 10^{5}$ 20 nm gold nanoparticles (GNP) and 1 particle per $4.97\times 10^{5}$ 40 nm GNP on one device. However, the atmospheric pressure measurement decreased NEMS performance and mass resolution. Consequently, measurements performed with their prototype on gold nanoparticles and viral particles exhibited substantial mass dispersion.

On the basis of literature reports, we hypothesized that there must exist a favorable operating pressure range that would allow reducing pumping requirements without affecting measurement performance. The goal of the present study was to establish this range through theoretical study and numerical simulation, an eventually demonstrate it experimentally. Ultimately, we applied our findings to develop a new NEMS-MS prototype characterized by decreased pumping requirements, superior focusing performances and improved particle capture efficiency over our previous system (Dominguez-Medina et al., 2018).

## 2 Materiel and methods

### 2.1 Working principle of NEMS-MS

**FIGURE 1:**
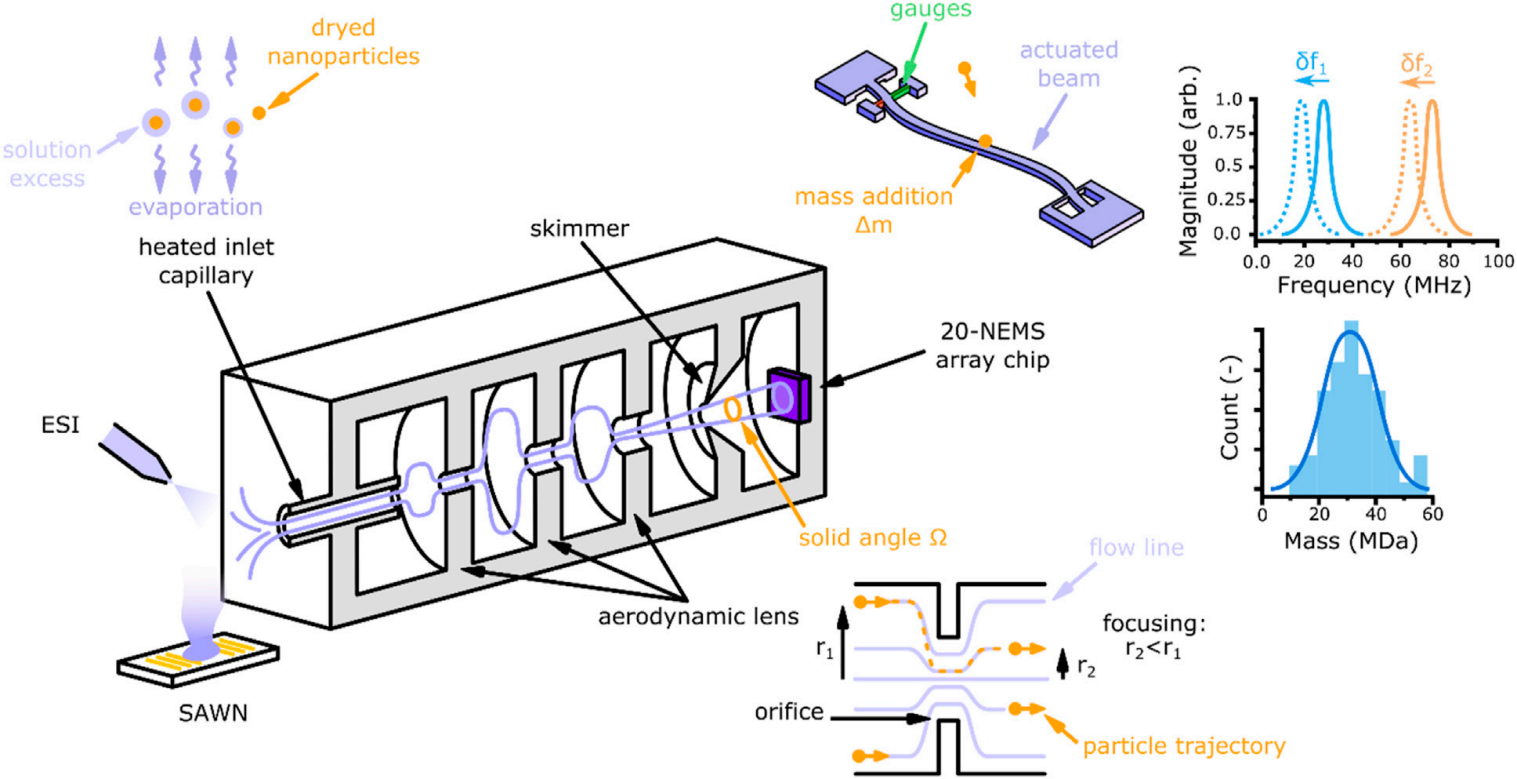
Overall scheme of NEMS-MS system architecture.

Along the development iterations, the core of the NEMS-MS architecture remained unchanged (see Figure 1). It consists of four main parts: a nebulization and desolvation stage, an aerodynamic focusing lens followed by a skimmer, and an array of sensing elements.

The NEMS-MS technique analyzes particles from the gas phase, and thus an aerosol must be generated when the analytes are in solution. Two aerosolization methods are typically used: Surface Acoustic Wave Nebulization (SAWN) and nano ElectroSpray Ionization (nESI). The two methods are suitable for biological samples (e.g. viruses, virus-like particles) in small volume (<ml) of buffer solutions. SAWN produces lower kinetic energy droplets, promoting particle sampling through the inlet capillary. Nanoparticles can also be generated using a constant output atomizer, in order to produce larger amount of aerosol required to test system transmission and focusing. The nebulized particle stream passes through a stainless-steel inlet capillary which can be heated up to 250°C in order to dry particles and prevent solvent influence on the mass measurement (Clement et al., 2021). The inlet capillary typically is 250 µm in diameter, and 11 cm long and drives pressure levels in the following chambers of the instrument. Once the solvent excess has been removed, the aerosol must be focused to optimize individual particles capture and detection by the nano-resonator array. An aerodynamic lens is used to perform inertial focusing, producing a narrow particle beam. The longitudinal shape of the particle beam being a cone, the particle number flux (particle/m^2^/s) decreases with the distance from the lens outlet. Consequently, one of the key parameters of the design is the lens-to-sensor distance, which shall be kept as small as possible. More details about the aerodynamic lens physical principle, simulation and characterization are provided in Section 2.3. Finally, the analyte stream reaches an array of 20 NEMS resonators fabricated from silicon on insulator (SOI) wafers using very large scale (VLSI) integration process (Mile et al., 2010). As the beams are 300 nm wide and their length is about 10 μm, their active area is exceedingly small. An analyte particle reaching a resonator’s active surface induces simultaneous shifts in its resonance frequencies, which can be directly related to the mass and position of the landed particle (Sage et al., 2018).

### 2.2 NEMS quality factor measurement

#### 2.2.1 Doubly clamped nano resonators

**FIGURE 2:**
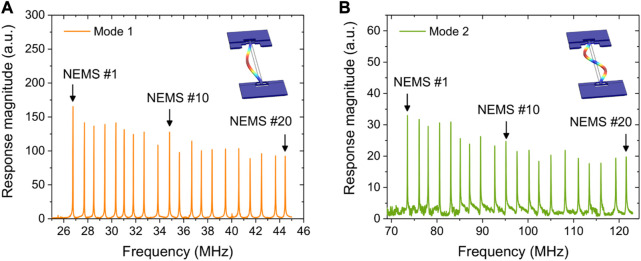
Frequency response of a 20 NEMS array for **(A)** mode 1 and **(B)** mode 2. Insets of each panel present the mode of vibration obtained using COMSOL simulation, and represented with exaggerated amplitudes for the sake of visualization. Each peak corresponds to the resonance of a single NEMS resonator. The length of resonators NEMS #1, NEMS #10, NEMS #20 are 9.04, 8.7, and 7.61 µm, respectively.

Each NEMS within the array comprises a doubly-clamped vibrating beam electrostatically actuated at its resonance frequency. Two piezoresistive nano gauges near one end of the beam compress and stretch due to the it’s displacement, generating a differential signal at the beam’s oscillation frequency (see Figure 1). The resonators composing the array were designed with different lengths (see Table 1) in order to be addressed using different resonance frequencies, as shown in Figure 2. They all are 160 nm thick and 300 nm wide and their respective lengths are reported in the Supplementary Material; Section 9.

**TABLE 1:** Length, resonance frequency and quality factors for 3 nano resonators.

NEMS ID	Length [µm]	Mode 1		Mode 2	
		f [MHz]	Q [-]	f [MHz]	Q [-]
1	9.04	26.75	4000	73.47	2200
10	8.07	34.80	3600	95.10	1900
20	7.61	44.45	3000	122.00	1700

Resonator-based MS measurements require retrieving the frequency information from each individual resonator. Initial resonance frequencies and phase references are recorded for every resonator in the array (*cf*. Figure 2). Then, a phase-locked loop (PLL) is locked onto a given resonator to monitor and register frequency data for two modes successively for a given period, called idling time $\tau_{PLL}$ , after which it switches to the next resonator. Any landing particle event results in a mass addition Δm and causes quasi-instantaneous shift in the n -th mode resonance frequency $\Delta f_{n}$ . The following relationship describes how $\Delta f_{n}$ and Δm are interrelated:

$$\Delta m=M\frac{\Delta f_{n}}{f_{n}}\frac{\alpha_{n}}{\phi_{n}^{2}\left(x\right)}$$ (1)

Where M is the total mass of the beam, $f_{n}$ the resonance frequency of the n -th mode, x the particle landing position, $\phi_{n}\left(x\right)$ the n -th mode shape and $\alpha_{n}$ a constant defined by $\alpha_{n}=-2\int_{x^{\prime }=0}^{x^{\prime }=1}\phi_{n}^{2}\left(x^{\prime }\right)dx^{\prime }$ . Equation 1 contains two unknowns: the particle mass Δm and its landing location x ; thus, a two-equations system must be solved. In practice, the nano-resonator beams are actuated at their first and second modes so the two variables can be derived. The frequency jumps are considered as actual particle landing events when the frequencies shift by amounts larger than 5 times the average frequency noise in each mode (*cf*. Supplementary Material; Section 5). As a consequence, a crucial parameter for particle mass determination is the frequency stability over the measurement period, which must be as high as possible at the time scale of $\tau_{PLL}$ .

#### 2.2.2 Characterizing NEMS for various pressures

The nano-resonators used as sensors can be described by several characteristics, one of which is the quality factor (Q factor), which captures information about energy dissipation in the system. The most significant dissipation for NEMS resonator operating in ambient conditions is caused by viscous damping (Li et al., 2007). To minimize energy losses and therefore maximize the quality factor of the sensor, NEMS are typically operated in a vacuum environment. However, it has been suggested that, as their critical dimensions approach the mean free path of gas molecules at ambient pressure, the Q factor of nanoscale devices is only marginally reduced while transitioning from high vacuum to atmospheric pressure (Li et al., 2007). Because our goal was to rationalize our system, one possible way entailed relaxing pumping requirements. To evaluate this possibility, we studied the effect of the pressure on NEMS resonance frequencies, their quality factor, and frequency stability. The experimental apparatus used to measure the relevant metrics (i.e., resonance frequency, Q factor and frequency stability) consisted in a vacuum chamber connected to a primary pump and equipped with a 925 micro-Pirani gauge. In order to vary the pressure inside the chamber, a valve was used to create a nitrogen leak allowing to perform measurements in the range $10^{-3}-10^{3}\;mbar$ .

### 2.3 Aerodynamic lens

#### 2.3.1 Inertial focusing

Aerodynamic lenses for aerosol sampling and focusing have been developed since the 1990s (Liu et al., 1995). They use particle drag force and inertia in order to manipulate particles through flow contractions induced by a series of orifices. This produces sudden changes in particle’s motion that result in radial particle shifts, for particles having relaxation time higher than the obstacle characteristic time. In aerosol physics, this phenomenon is often characterized using the Stokes number, which is the ratio between particle’s inertia and the drag force to which they are subjected, given by:

$$St=\frac{\rho_{p}d_{p}^{2}C_{c}\left(d_{p}\right)u}{18\eta L}$$ (2)

Where $\rho_{p}$ is the particle density, $d_{p}$ its diameter, u the fluid velocity and $C_{c}$ the Cunningham correction slip coefficient used to correct the drag force expression as a function of the flow regime (molecular, transition, continuum). η is the fluid viscosity and L is the obstacle characteristic length. When the Stokes number is close to unity, particles deviate from their trajectory due to their inertia but are quickly reattached to a flow line closer to the axis than their previous radial location. This describes how particles should behave when passing through one aerodynamic lens orifice. Since the Stokes number is a function of particle diameter and mass, an aerodynamic lens usually consists of several orifices focusing different particle sizes. Forcing particles to pass through multiple orifices therefore results in a narrower particle beam for a range of particle sizes.

#### 2.3.2 Numerical model

Several authors modelled how nanoparticles behave within an aerodynamic lens (Zhang et al., 2002; Wang et al., 2005a; Abouali et al., 2009). A common approach is based on lagrangian tracking of the particles through the aerodynamic lens, ultimately allowing computation of the particle beam solid angle. COMSOL Multiphysics was used to solve steady, compressible, viscous, laminar Navier-Stokes equations. For computation efficiency’s sake, an axisymmetric geometric domain was used. When it comes to boundary conditions, no slip boundary was used for walls, and both the downstream pressure and the throughput mass flow were computed using a macroscopic vacuum system model (cf. Supplementary Material; Section 3). As for the lagrangian part of the simulation, two forces were taken into account: the drag force and the Langevin force used to model brownian motion. The analytical formulations of both forces feature the Cunningham correction slip factor, depending itself on the flow regime (i.e., the Knudsen number) which had to be updated due to the pressure gradient particles are travelling through. The physical model was solved using the velocity Verlet algorithm with an adaptative time step and was written in Python.

#### 2.3.3 Characterization

First, the aerodynamic lens focusing ability was characterized based on polystyrene nanoparticle deposition on a silicon target located at the same position than the detector. The polystyrene 100 nm nanoparticles colloidal solution (Magsphere, Pasadena, California, United States) concentration was $1\times 10^{11}$ particles/ml and was nebulized using nESI at a flowrate of 8 μl/min for 30 min. The inlet capillary was heated at 85°C. In order to estimate the diameter of the deposition pattern, the target was observed using binocular loupe and the resulting picture was processed by analyzing pixel intensity profiles (*cf*. Supplementary Material; Section 7).

The second approach aimed at validating the transmission efficiency of the focusing lens by using the NEMS sensors, and comparing first and second NEMS-MS prototypes characteristics. In this approach, three gold nanoparticles colloidal solutions were investigated. 20 and 40 nm diameter GNP were purchased from BBI Solutions (Crumlin, Wales, UK) and the nm diameter GNP were purchased from Sigma Aldrich (Saint-Louis, Missouri, United States). GNP were sprayed using SAWN or nESI. The mass spectra—Converted into geometric diameter—And event counts per minute and per active NEMS devices were evaluated.

### 2.4 Pumping requirements

As our overall objective was to decrease the pumping requirements, the design phase included a pressure calculation step. A mass conservation model (cf. Supplementary Material; Section 3) was used to compute the pressure in each chamber based on a given pumping speed. It helped finding design parameters (inlet capillary length and diameter, lens orifice diameters, skimmer diameters) matching NEMS and aerodynamic lens compatible pressure ranges. This model was a keystone in the iterating process for simulating several pumping setups. Importantly, the optimal pressure range for pump performance was also taken into account, as turbomolecular pumps capacity steeply degrades below $10^{-3}$ mbar.

## 3 Results and discussion

One of the key parameter to downsize NEMS-MS prototype was the operating pressure: turbo-molecular pumps are large and heavy and require cumbersome vacuum chambers and frame. However, limiting the pressure is necessary to avoid the nano-resonator dampening leading to degradation of the quality factor and thus poorer mass resolution. Furthermore, the operating pressure of the aerodynamic lens, linked to a large extent to the analytical throughput, also plays a role in the quality of the inertial focusing. As a consequence, both of these aspects had to be addressed independently in order to determine the suitable operating pressure range.

### 3.1 Nano-resonators in moderate vacuum

We studied the dependence of the quality factor (Q factor) of several resonators with respect to the operating pressure. In order to cover the various beam dimensions from our 20 resonators arrays, we selected three different beams (i.e. the ones with smallest, largest and median length) and characterized their mechanical parameters—Resonance frequency, quality factor, and frequency stability—As a function of pressure.

**FIGURE 3:**
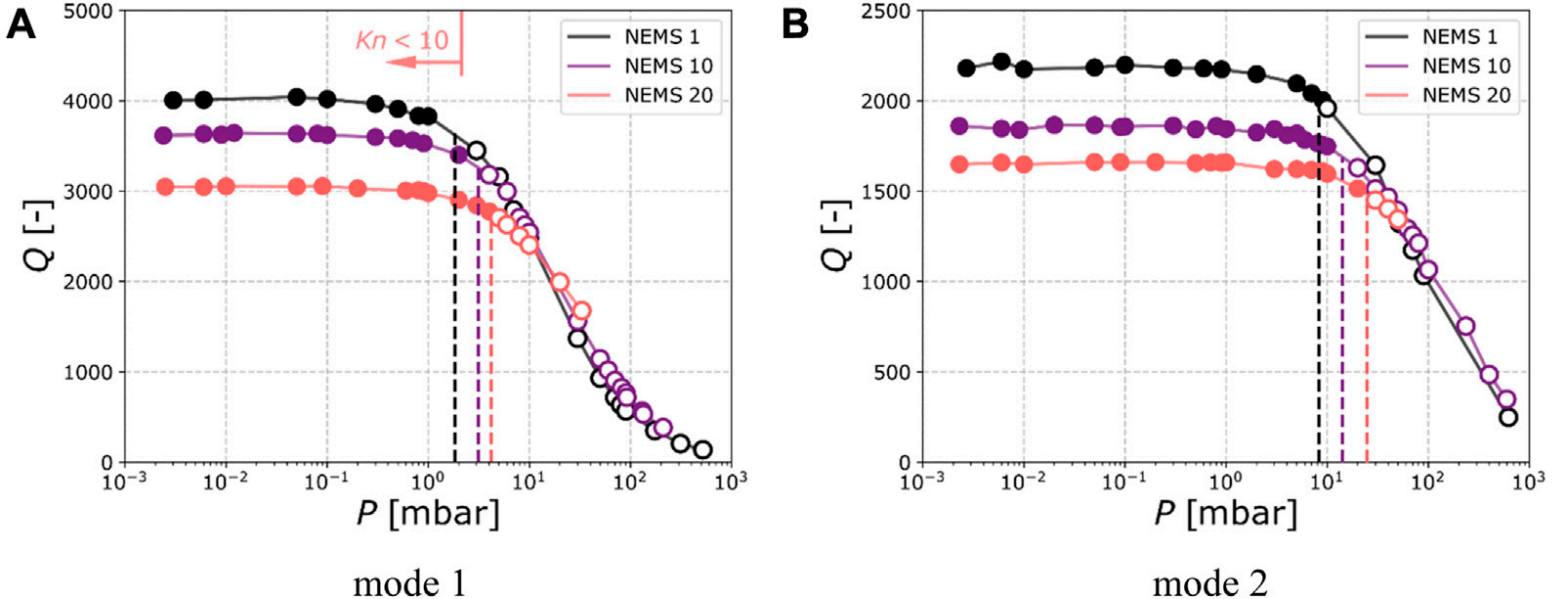
Resonance quality factor of the first **(A)** and second **(B)** modes for the NEMS number 1, 10 and 20 when operated in at various pressures. This three NEMS have different lengths and thus different resonance frequencies (see Table 1). The dashed vertical lines indicate pressures at which the quality factor loses 10% of its maximum value.

Figure 3 displays the Q factors as a function of operating pressure for the three selected resonators. Before reaching high pressures, Q factors are constant and are, for resonators 1, 10 and 20: 4000, 3600 and 3000 for the first mode. For the second mode, their values are 2200, 1900, and 1700, respectively. A loss of 10% of the maximum value (highlighted by dashed lines) was observed at 2, 3, and 4 mbar for the first mode and at 10, 20, and 25 mbar for the second mode for NEMS number 1, 10 and 20 respectively. The trend presented by quality factor as a function of pressure has been previously reported (Li et al., 2007; Gavan et al., 2009), and our results confirm prior observations for our specific devices. A model based on the hydrodynamic function with comparisons to other models was described in (Aoust et al., 2015). The manifestation of the minor effect of the pressure on the quality factor can be given by the flow regime. It can be determined by the Knudsen number $Kn=\lambda /L_{c}$ , which is the ratio between the molecule mean free path λ and a resonator characteristic dimension $L_{c}$ . In our case, the width of the beams (300 nm) can be considered to estimate the Knudsen regime (Gavan et al., 2009). If the mean free path λ is larger than $L_{c}$ (when Kn>10 ), the probability that the beam encounters a gas molecule is small, leading to a null viscous damping. In this regime, the damping is mainly caused by thermoelastic effects and energy transfer into the support (clamps). In our case, the most significant losses are connected with energy dissipation into the support (cf. Supplementary Material; Section 6). Viscous damping is introduced for a Knudsen number Kn<10 , leading to decrease of the quality factor. The vertical line on the graph Figure 3A was plotted to delimit the relevant Knudsen regimes. For devices with characteristic dimensions in the hundreds of nanometers, this regime shifts toward higher pressure values, and the operating pressure may be increased without sacrificing performance.

Another parameter that can determine the flow regime is the Weissenberg number Wi=τ/T , which is a ratio of the characteristic time scale T to the relaxation time τ in the medium. It has been shown (Karabacak et al., 2007) that increasing the resonance frequency of nano-resonators allows to reach the molecular flow regime where viscous damping becomes negligible. This may explain why, for the second mode of vibration, the quality factor was less influenced by a comparable increase in pressure than the first mode. Moreover, for the same mode of vibration, smaller resonators (i.e. having a higher frequency) are characterized by a lesser quality factor reduction as a function of pressure, which was also consistent with previous observations (Karabacak et al., 2007).

**FIGURE 4:**
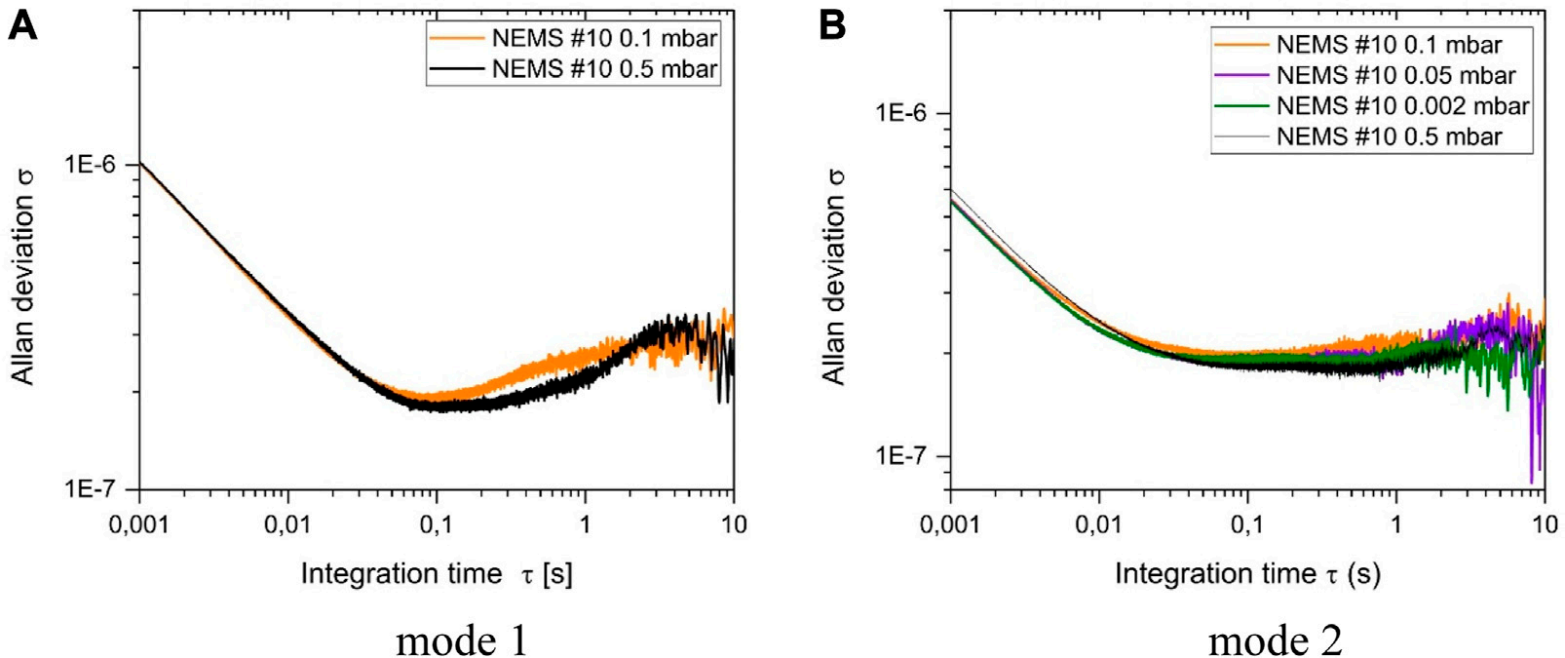
Allan deviation of the first **(A)** and second **(B)** modes for the NEMS number 10.

Frequency stability is a key performance parameter for nano-resonators used in mass spectrometry as the particle masses are deduced from frequency shifts. Lower frequency stability causes higher noise level and degraded mass resolution. The frequency stability was characterized using Allan deviation and was measured as a function of pressure from the closed loop frequency trace of an individual resonator. We show that this parameter remains constant until a pressure of ∼ 0.5 mbar (cf. Figure 4), showing that mass resolution and hence measurement performance remain stable up to this value. Other Allan deviation plots are reported in the Supplementary Material; Section 1.

Our investigations showed that our nano-resonators could operate in a pressure regime as high as 0.5 mbar without sacrificing mass resolution. Moreover, they confirmed that using smaller NEMS would allow further reduction of the pumping requirements and thus limit the apparatus footprint.

### 3.2 Aerodynamic focusing

The ability to operate nano-resonators at a relatively high pressure (or low Knudsen number) is the foundation of the NEMS-MS prototype downsizing. Yet, another important aspect of the instrument depends on pressure: particle transport to the NEMS detector. Therefore, we had to also validate the operation of the aerodynamic lens at higher pressure.

The first NEMS-MS prototype (Dominguez-Medina et al., 2018) integrated an aerodynamic lens which was designed using (Wang et al., 2005b) guidelines. It was optimized to collimate virus-like nanoparticles of diameter in the 100 nm range and unit density. It was experimentally characterized with 45 nm polystyrene particles at an upstream pressure of 10^2^ mbar and a downstream pressure of 10^−2^ mbar. The resulting deposit at the NEMS location was measured to be 1.5 mm diameter. Since this performance matches the targeted NEMS-MS applications, we decided to retain the same aerodynamic lens design and investigate its focusing abilities at higher pressure.

As described earlier, the selected approach was based on the particle lagrangian tracking through the aerodynamic lens because it could directly provide the particle beam solid angle. Boundary conditions, including mass flow and downstream pressures, were computed using the vacuum system model (cf. Section 2.4; Supplementary Material; Section 3). Ultimately, calculated pressures were compared with actual measurements and found to be in good agreement.

**FIGURE 5:**
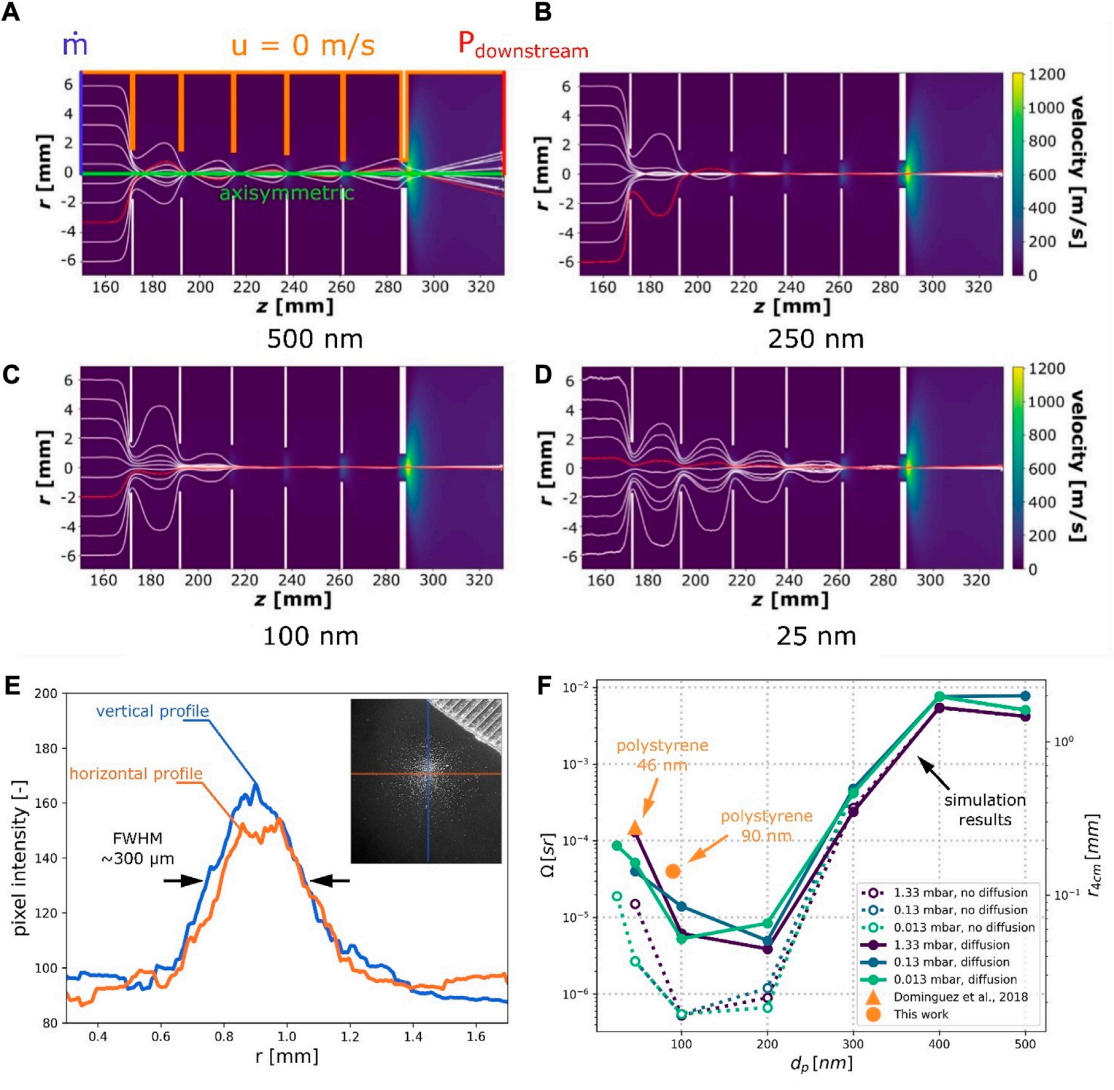
Particle trajectories through the lens simulated for different particle diameters: **(A)** 500 nm, **(B)** 250 nm, **(C)** 100 nm and **(D)** 25 nm. **(E)** Pixel intensity profiles of the deposit photograph along two axes, located by the orange and blue lines on the inset. **(F)** Solid angle Ω and particle beam radius at 4 cm downstream the lens (location of the NEMS sensor) computed for several downstream pressures. Orange points represent experimental data and dots are simulation results.

Figures 5A–D present particles trajectories inside the aerodynamic lens computed with an exit pressure of 1.33 mbar for particles of different diameters and a density of 1.06 g/cm^3^, which corresponds to polystyrene and is close to that of viral samples for which the lens was initially designed. Based on these results, both the solid angle Ω and the particle beam radius 4 cm downstream the aerodynamic lens (written $r_{4cm}$ ) were derived for different particle diameter $d_{p}$ . The 4 cm distance corresponds to the location of the NEMS array in the new mechanical architecture. The results reported in Figure 5F show how the solid angle behaves depending on particle diameter and that the solid angle remains optimal for the range 100−200 nm . Moreover, the addition of the Langevin force used to model diffusion plays a significant role only for particles smaller than 300 nm and yields more realistic predictions when compared to experimental results (orange dots). Finally, within the investigated pressure range ( $1.33\times 10^{-2}-1.33$ mbar), the model showed that the impact of diffusion remained relatively constant and pressure had a limited influence on the resulting particle beam’s solid angle.

We verified the numerically computed focusing of the aerodynamic lens by exposing silicon targets to a ∼90 nm average diameter polystyrene nanoparticle beam. Using a binocular loupe and image processing, the deposit diameter was estimated to be approximately 300 μm (*cf*. Figure 5E). This value was reported on Figure 5F and is consistent with the numerical model predictions. It is worth noting that this represents a factor 5 improvement over the previous system generation (Dominguez-Medina et al., 2018) in terms of beam diameter. This is mainly due to the smaller lens-to-detector distance which used in the novel design (4 *vs*. 8 cm). According to these results, as for the NEMS sensor, the aerodynamic lens may be used without performance degradation over the studied pressures for the size range of interest, confirming that the NEMS-MS architecture is compatible with a substantial reduction of the pumping system.

### 3.3 Compact and modular NEMS-MS design

Following the aerodynamic lens study and the NEMS performance analysis over a wide pressure range, the vacuum system was modelled as described in Section 2.4 and a new pumping strategy was defined to maintain a 0.1 slm mass flow and related pressures of 2.7 mbar downstream the lens and ${1.3\times 10}^{-2}$ mbar inside the sensor chamber. Instead of the two turbomolecular pumps ( 450 l/s and 250 l/s) used to pump the system downstream of the aerodynamic lens and in the sensor chamber respectively, a single smaller turbo-molecular pump (TwisTorr 74FS, Agilent Technologies, Les Ullis, and France) was used to pump down the sensor chamber. A backing primary scroll pump (IDP-3, Agilent Technologies, Les Ullis, and France) was also used to pump the chamber downstream of the aerodynamic lens and back up the turbo-molecular pump.

**FIGURE 6:**
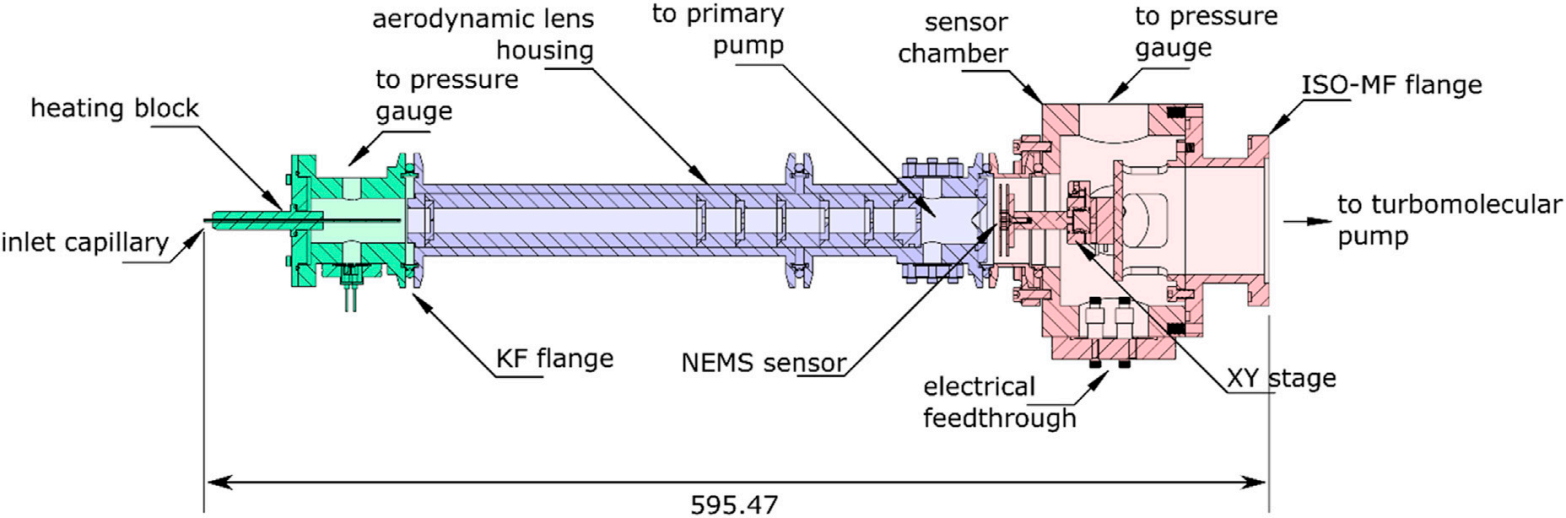
Mechanical compact and modular design of the NEMS-MS prototype. The green parts are the inlet section, the blue parts comprise the aerodynamic lens and the skimmer. The red parts are the sensor chamber, designed to hold the turbo molecular pump and the XY actuated stages.

One of the objectives underlying the reduced pumping is to downsize the NEMS-MS bench in order to make it more practical to use. Thus, this second-generation prototype was engineered considering not only the dimensions, but also the modularity of the instrument. This will allow future users facing novel challenges to upgrade the instrument. Therefore, each function (particle intake, aerosol focusing and particle mass measurement) has been attributed a different mechanical module, as shown on Figure 6. Having opted to limit the diameter of the instrument, the mechanical cohesion of the assembly could be ensured by KF50 flanges, which are easier to mount, open and close. Moreover, the overall system was mounted on a rail, keeping the sensor chamber fixed and allowing the inlet chamber and the aerodynamic lens to slide away from the nano-resonator housing to access and replace the sensor’s chip.

Because of the heavy pumps operated on the first-generation prototype, heavy vacuum components were used to support the weight and to prevent mechanical vibrations. Large hardware parts made the aerodynamic lens-to-detector distance optimization difficult and constrained the sensing area to deposit area ratio. Moreover, large mechanical parts being complex and expensive to machine, a built-in alignment solution could not be implemented, and a port aligner as well as a XY stage were used to align the lens outlet and the NEMS array respectively. As a result, one of the most time-consuming tasks to perform when replacing a NEMS chip was the alignment process, which was required to keep the sensor at the center of the particle beam produced by the aerodynamic lens. This operation was facilitated in the new system by designing a native alignment between the inlet capillary, the aerodynamic lens housing and the skimmer. Henceforth, the alignment step reduces to positioning the sensor within the particle beam area instead of aligning multiple parts aerodynamic lens and sensor as in the previous NEMS-MS prototype.

Finally, since the novel NEMS-MS prototype may host various sensor technologies—Nano-electromechanical as well as nano-optomechanical devices—The sensor chamber was designed accordingly, guaranteeing an easy and compact alignment solution whatever the type of chip used. The sensor itself is packaged onto a printed circuit board which is mounted on a motorized XY stage (Agilis AG-LS25, Newport, Irvine, California, United States), which could be used to monitor the sensor location and scan the particle beam.

### 3.4 Gold nano particles mass spectra

**FIGURE 7:**
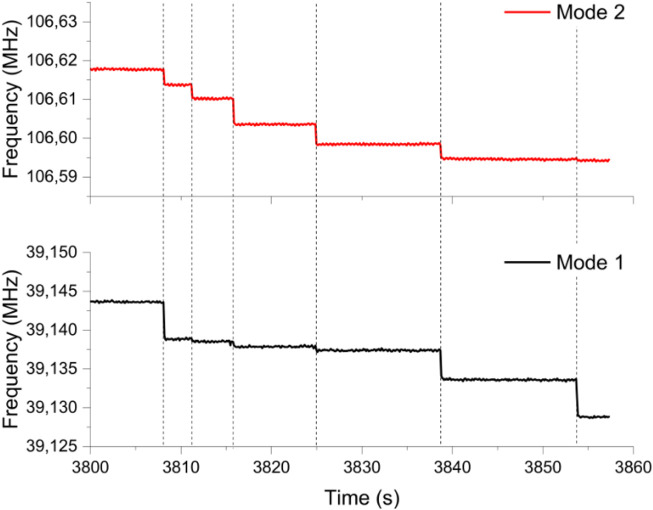
Phase-locked loop raw frequency traces for one resonator as a function of time during the deposition of 20 nm GNP produced by nanoESI. Black and red lines represent first and second mode, respectively. Dashed lines indicate mass deposition events.

**FIGURE 8:**
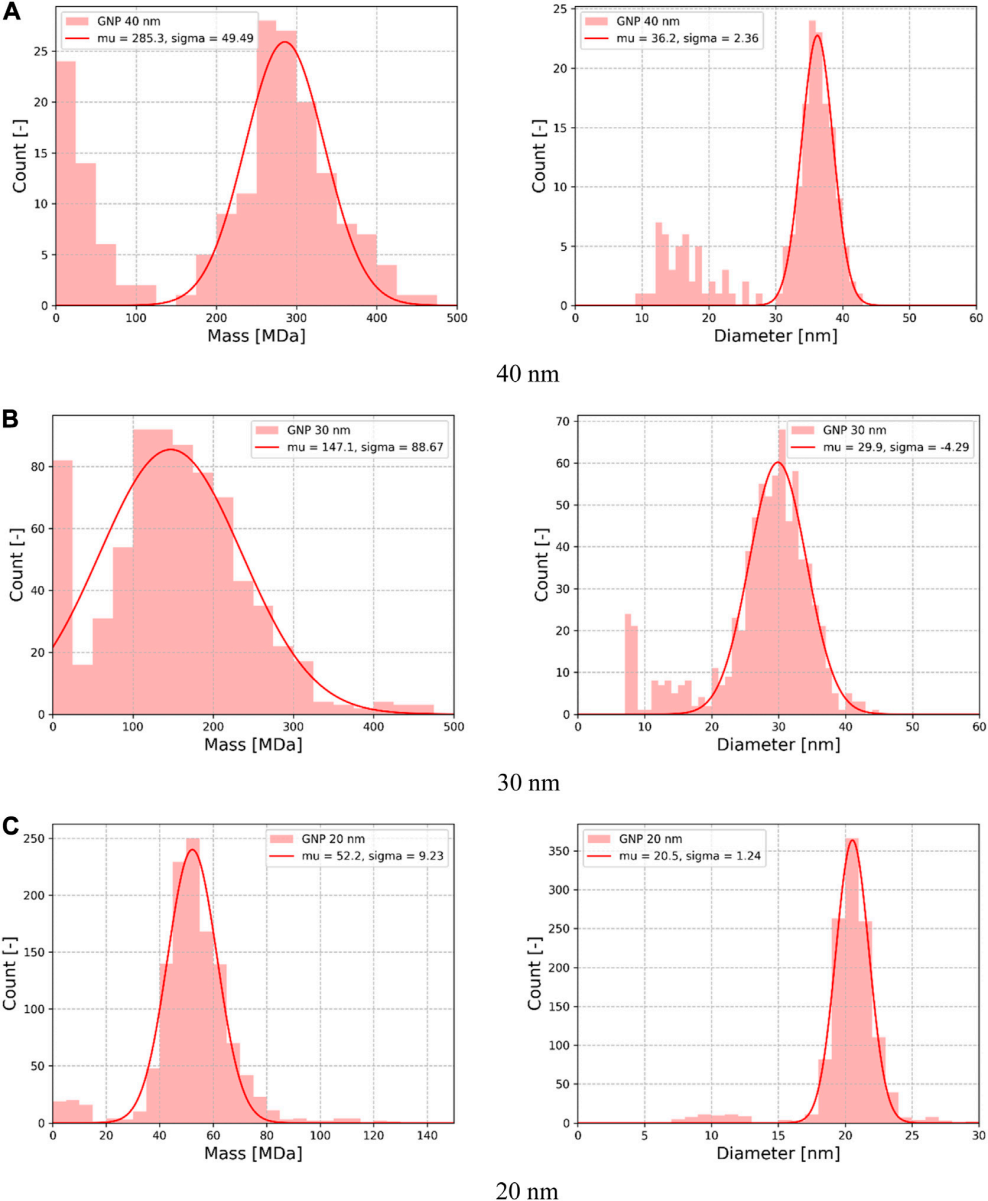
Histogram of the mass measurement performed during 60 min of acquisition time retrieved from the frequency shifts for gold NP of diameter **(A)** 40 nm **(B)** 30 nm **(C)** 20 nm.

Eventually, to assess the new-generation compact and modular NEMS-MS system, we performed mass analysis of a series of calibrated samples of GNP of sizes 20, 30, and 40 nm. Nanoparticle colloidal suspensions were diluted in methanol, achieving concentrations of $4.50\times 10^{10}$ to $3.50\times 10^{11}$ particle/ml depending on nanoparticle diameter. Figure 7 presents examples of frequency traces obtained in both resonance modes during exposition to 20 nm GNP. The amplitudes of the observed frequency discontinuities can be translated into the mass-position domain, allowing the production of a particle mass distribution (PMD) (cf. Section 2.2.1). The distributions obtained for the three sampled analyzed in this study are shown in Figure 8.

Since GNP manufacturers characterized the batches by measuring nanoparticle diameters, the PMD were translated into mass equivalent diameters, assuming perfect sphericity and homogeneous density equal to that of bulk gold ( $\rho_{gold}=19.3\;g/{cm}^{3}$ ). Gaussian functions were used to fit masses and diameters histograms. Fitting parameters are reported in Table 2 and show good agreement with manufacturer’s data (diameter measured by SEM), the relative difference in size ranging from 0.3% to 16% depending on the sample, and from 8.0% to 11.9% in mass. The standard deviation also compared well with manufacturer’s data. Moreover, it should be highlighted that even if this comparison allowed to quantify the difference between NEMS-MS mass measurement and SEM size measurement, these techniques do not determine the same metric. Indeed, in order to compute the diameter of a particle, SEM is based on 2D image processing and NEMS-MS performs a mass-to-size conversion. Thus, both techniques assume the sphericity of particles, but the different metrics may induce different error factors. Finally, one might keep in mind that nano-resonator mass measurement has its own uncertainty as investigated previously by Clement et al. (2021).

**TABLE 2:** Gaussian fit parameters (mean and dispersion) compared with manufacturer data.

	Mass			Diameter		
Sample	NEMS-MS $\bar{m}$ ( $\sigma_{m}$ )	SEM $\bar{m}$	Relative difference	NEMS-MS $\bar{d_{p}}$ ( $\sigma_{d_{p}}$ )	SEM $\bar{d_{p}}$	Relative difference
	[MDa]	[MDa]	[–]	[nm]	[nm]	[–]
20 nm (BBI)	52.2 (9.2)	46	11.9%	20.5 (1.2)	19.6 (<1.6)	4.4%
30 nm (Sigma-Aldrich)	147.1 (88.7)	164	11.5%	29.9 (4.3)	30 (2)	0.3%
40 nm (BBI)	285.3 (49.5)	308	8.0%	36.2 (2.4)	37–42 (N/A)	2.2%–16%

In addition to characterizing mass measurement accuracy and precision, these experiments allowed to estimate the particle capture efficiency, which is useful for comparison with the previous system. Particle capture efficiency, as defined as the ratio between the number of detected particles to the number of GNP nebulized from solution during the measurement, was determined for one single NEMS among the 20 resonators of the array. The measurement details (i.e., flow rate, duration, initial concentration) are presented in the Supplementary Material (Section 8). The particle capture efficiencies were computed for each GNP sample and are reported in Table 3. These values are compared with those reported by Dominguez-Medina et al. (2018), performed with ESI nebulization technique, showing an improvement factor of 20–200. The detection efficiency improvement could partly be attributed to the reduction in aerodynamic lens-to-sensor distance which has been halved (4 cm *vs*. 8 cm). Another possible reason may be that the new prototype is less affected by pump vibrations as the lens housing and the skimmer are mounted contiguously. Moreover, the lens housing is also aligned with the rest of the mechanical parts of the system, and has no degrees of freedom. These mechanical optimizations indirectly benefited from the pumping requirements downsizing, which allowed to reduce every dimension of the system, and were not possible on the first-generation prototype due to the cumbersome vacuum hardware components.

**TABLE 3:** Capture efficiency computed for the gold nanoparticles. ^1^Range computed based on the upper and lower capture efficiency reported by (Dominguez-Medina et al., 2018), namely 1 per ∼2×10^9^ and 1 per ∼1×10^10^ sprayed particle, respectively. ^2^ Capture efficiency expressed as 1 detected particle per N nebulized particles.

GNP sample	Sprayed particles	Number of NEMS	Event count	Capture efficiency^2^	Capture efficiency for 1 NEMS^2^	Improvement factor^1^
	[ $\times 10^{9}$ NP]	[-]	[-]	[-]	[-]	[-]
20 nm (BBI)	12.3	9	1220	1 per $4.5\times 10^{6}$	1 per $9.0\times 10^{7}$	22–111
30 nm (Sigma-Aldrich)	3.12	12	739	1 per $2.5\times 10^{6}$	1 per $5.1\times 10^{7}$	40–198
40 nm (BBI)	1.58	9	183	1 per $3.9\times 10^{6}$	1 per $7.8\times 10^{7}$	26–129

The interest in analyzing large sample fractions grows when the amount of available sample volume is small, which is often the case for biological samples, or for broad mass distributions that require a lot of events to acquire adequate statistics. To pursue the same objective (Erdogan et al., 2022), chose to dispense with vacuum system altogether and performed on-chip electrostatic focusing at ambient pressure. The main benefit of this technique was to reduce the volume and the cost of the apparatus while achieving a particle capture efficiency of 1 per $1.85\times 10^{5}$ particle to 1 per $4.97\times 10^{5}$ particle for one device. At this point, it should be stressed that particle capture efficiency comparisons are complicated due to potentially different raw resonance frequency postprocesses.

## 4 Conclusion

In order to downsize the first generation NEMS-MS prototype without compromising mass measurement quality, the impact of an increase in operating pressure was analyzed. For this purpose, we separately addressed the NEMS and the aerodynamic lens. NEMS devices were characterized experimentally over a wide pressure range ( $10^{-3}$ – $10^{3}$ mbar) and it was shown that their performance was unaffected at pressures up to 0.5 mbar. The aerodynamic lens was simulated numerically over an operating downstream pressure range of $1.3\times 10^{-2}$ – 1.3 mbar for particle within the range of 25–500 nm and did not show to be significantly affected by this parameter.

Based on these results, a new vacuum system has been designed with a single turbomolecular pump (74 L/s), replacing the two larger pumps used in the previous-generation assembly (450 L/s and 250 L/s). This radical optimization led to a more compact, lightweight, modular and easy to operate mechanical design. Moreover, it allowed to position the NEMS sensor closer to the aerodynamic lens outlet, improving the particle transfer from the source to the sensor. Performance improvement was quantified using two approaches: the particle beam area at the sensor has been measured to be approximately 5 times smaller than in the previous design, and the particle capture efficiency has been increased by a factor of 20–200. The prototype, based on arrays of NEMS operated with a frequency-addressing scheme, was validated with mass measurements of standard nanoparticle samples and showed excellent agreement with predicted values.

Particle capture efficiency being one of the main limitations of NEMS-based MS, it was one of the main motivations underlying this design optimization. Improving it allows to perform analysis faster: the data used to plot the spectra reported on Figure 8 were registered during 60 min experiments and would have required more than 10 hours on the previous setup. Overall, the NEMS-MS technique presents a trade-off between particle capture efficiency and mass resolution: increasing the NEMS exposition to aerosol seems to imply an increase in pressure, which in return affects the mass resolution. In our system, the trade-off to keep a 0.1 MDa mass resolution (Clement et al., 2021) seems to be, pressure-wise, at 0.5 mbar (cf. Figure 3). However, this threshold might not be suitable for smaller particles as the diffusion could cause the divergence of the particle beam downstream the aerodynamic lens (Wang and McMurry, 2006).

Although the presented NEMS-MS prototype yielded significantly enhanced performance, there is still potential for improvement. For instance, the size of the aerodynamic lens can be decreased, as it is the longest component in this prototype. Moreover, due to pumping considerations, the actual NEMS operating pressure ( $1\times 10^{-2}$ mbar) is still lower than the identified limit of operation ( $5\times 10^{-1}$ mbar), which opens the way to further developments using even smaller pumps or inlets with larger intake. Furthermore, detection efficiency could be further increased by NEMS and aerodynamic lens architecture, while sensitivity and mass range could be enhanced by NEMS sensor geometry. For the latter purpose, nano-optomechanical sensor might be implemented in this apparatus, which offer larger sensitive area and mass determination independently of particle landing position and physical properties (stiffness, size, and shape) (Sansa et al., 2020). These optimizations will eventually lead to new iterations of this NEMS-MS prototype, which will be greatly facilitated by its modularity.

## Data Availability

The raw data supporting the conclusion of this article will be made available by the authors, without undue reservation.
