# Supplementary material for: Compact and modular system architecture for a nano-resonator-mass spectrometer
Source: Front Chem. 2023 Sep 7;11:1238674. doi: 10.3389/fchem.2023.1238674 (PMC10569461; doi:10.3389/fchem.2023.1238674)
Supplement: Supplementary file 1 [file DataSheet1.pdf]

Supplementary Material

Compact and Modular System Architecture for a Nano-Resonator-Mass Spectrometer

Adrien Reynaud[1], Wioletta Trzpil[1], Louis Dartiguelongue[2], Vaitson Çumaku[2], Thomas Fortin[2], Marc Sansa[1], Sébastien Hentz[1], Christophe Masselon[2*]

*** Correspondence:** Christophe Masselon christophe.masselon@cea.fr

1 NEMS resonator characterization

1.1 Setup

The study of the relationship between the quality factor and the pressure was performed on a setup consisting of a 925 Micro Pirani vacuum gauge, a primary pump, a leak valve connected with a Teflon pipe to nitrogen. A lock-in amplifier (Zurich Instrument, Zurich, Switzerland) and a data software acquisition were used to study the resonance of the doubly-clamped nano-resonators.

1.2 Measurement

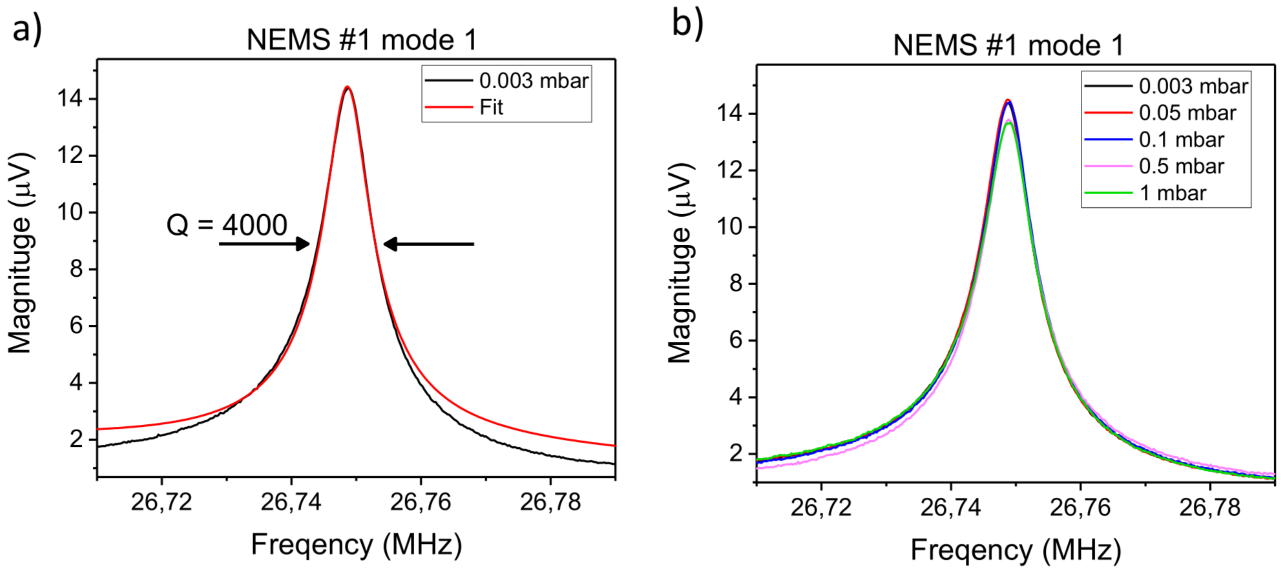

Supplementary Figure 1. a) Frequency response recorded for the mode 1 of NEMS #1 at pressure 0.003 mbar (black curve) with Lorentzian fit (red curve). b) Frequency responses recorded for the mode 1 of NEMS #1 at various pressures.

The quality factor Q was calculated from the fit of nano-resonator frequency response such as $Q = f_0/FWHM$, where f_0 is the resonance frequency and $FWHM$ is full width at half maximum. An example of measurement fitting is shown in the **Supplementary Figure 1**.

The **Supplementary Figure 2** presents the Allan Deviation of NEMS #1 and #10 for both modes of vibration and for various pressures. Frequency fluctuations seem to be constant within the pressure range under study.

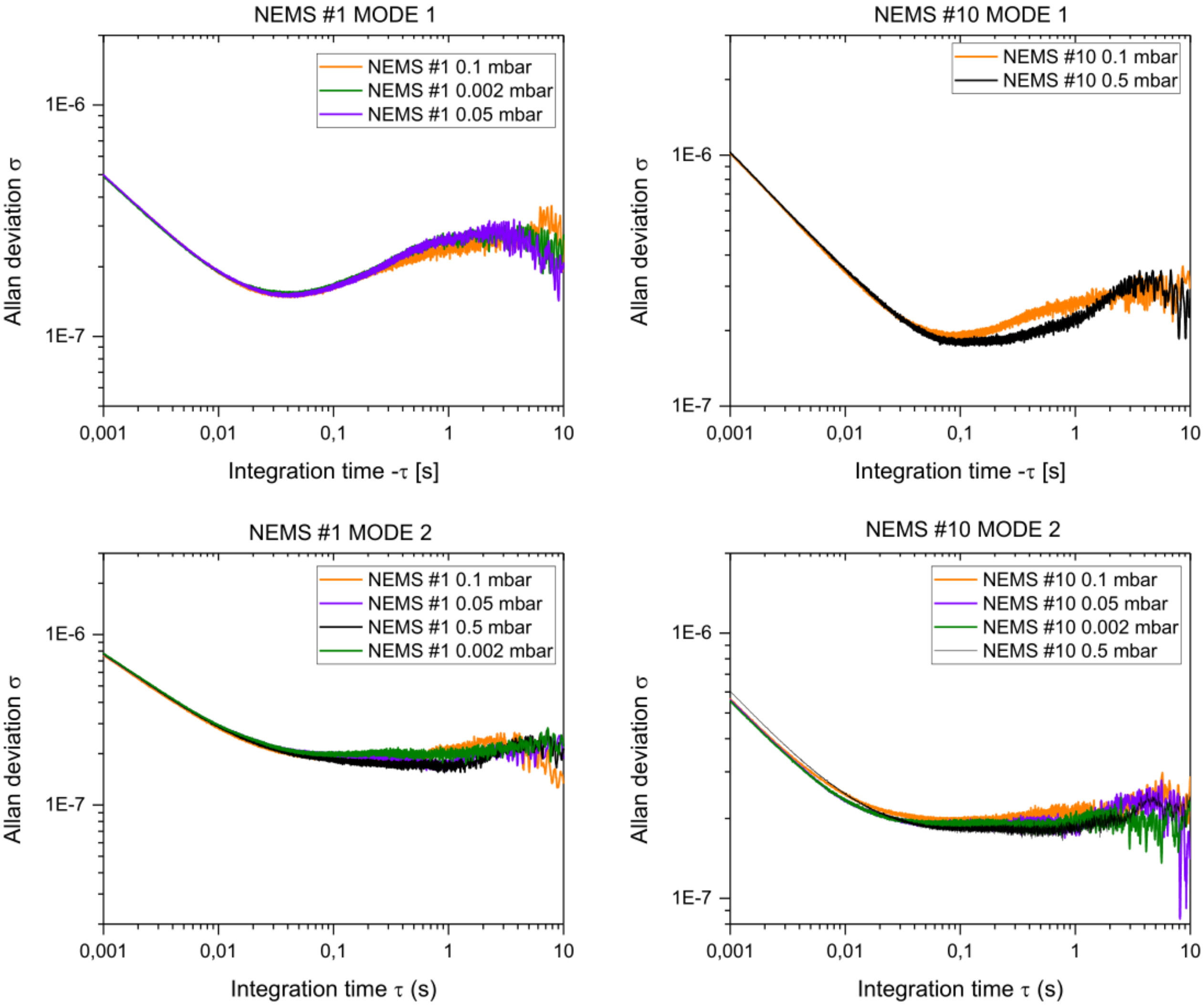

Supplementary Figure 2. Allan deviation measured for NEMS 1 and NEMS 10 at various pressures.

1.3 NEMS dimensions

Supplementary Figure 3 shows the SEM micrograph of the devices used in this work. A sketch presenting the dimensions of the system is also reported and the lengths of the beam are reported in **Supplementary Table 3**.

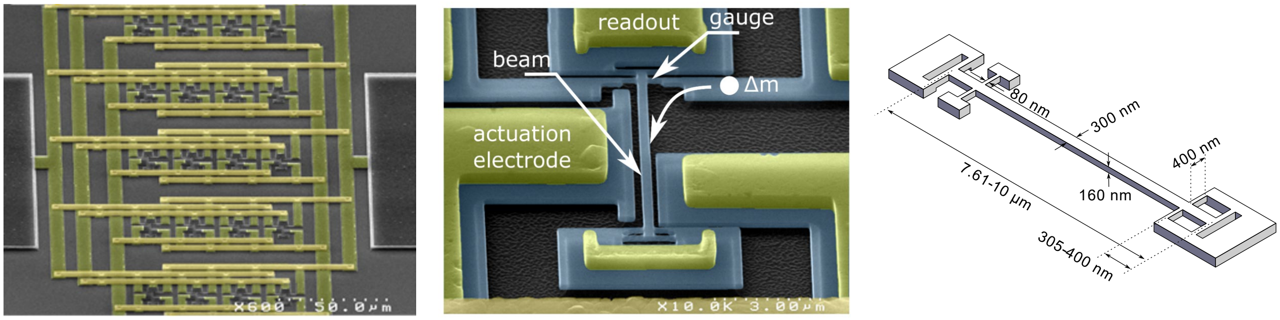

(a) SEM micrograph of the NEMS array

(b) SEM micrograph of a single resonator

(c) NEMS dimensions

Supplementary Figure 3. SEM micrographs and dimensions of the nano-resonators used in this work.

Supplementary Table 1. Lengths of every resonator beam of the array.

**NEMS index** [-]	**NEMS length** [µm]	**NEMS index** [-]	**NEMS length** [µm]	**NEMS index** [-]	**NEMS length** [µm]	**NEMS index** [-]	**NEMS length** [µm]
1	10	6	9.235	11	8.58	16	8.01
2	9.835	7	9.095	12	8.46	17	7.905
3	9.68	8	8.96	13	8.345	18	7.805
4	9.53	9	8.83	14	8.23	19	7.705
5	9.38	10	8.705	15	8.12	20	7.610

1.4 Mass resolution

Mass resolution σ_m is an important parameter for mass spectrometry and for NEMS-MS technique, it depends on the resonator geometry and the frequency fluctuations. It is defined as:

$$\sigma_m = 2M_{eff}\sigma_{\delta f} \tag{S9}$$

Where M_{eff} is the effective mass of the resonator and $\sigma_{\delta f}$ is the frequency fluctuation which can be measured using the Allan deviation. In our case, for 0.1 second integration time, its typical value is ranging from $\sigma_{\delta f} = 1 \times 10^{-7}$ to $\sigma_{\delta f} = 3 \times 10^{-7}$. Mass resolution has been computed for every NEMS and the results are reported in **Supplementary Table 2**. It slightly varies – depending on the length of the beam – between 0.12 MDa to 0.16 MDa for mode 1 and between 0.14 MDa to 0.18 MDa for mode 2.

Supplementary Table 2. Mass resolution of every NEMS of the array.

**NEMS index**	**Mass resolution**	**NEMS index**	**Mass resolution**	**NEMS index**	**Mass resolution**	**NEMS index**	**Mass resolution**

[-]	[MDa]	[-]	[MDa]	[-]	[MDa]	[-]	[MDa]
1	0.16/0.18	6	0.15/0.16	11	0.14/0.15	16	0.13/0.14
2	0.16/0.17	7	0.14/0.16	12	0.14/0.15	17	0.13/0.14
3	0.16/0.17	8	0.14/0.16	13	0.13/0.15	18	0.13/0.14
4	0.15/0.17	9	0.14/0.16	14	0.13/0.15	19	0.12/0.14
5	0.15/0.17	10	0.14/0.15	15	0.13/0.14	20	0.12/0.14

2 Aerodynamic lens

2.1 Dimensions

Dimensions of the aerodynamic lens are presented in Supplementary Table 3. They also correspond to the lens used and characterized by (Dominguez-Medina et al., 2018).

Supplementary Table 3. Aerodynamic lens dimensions. $D_{orifice}$ corresponds to the orifice diameter of each lens, L_{spacer} corresponds to the distance between the lens i and the lens i+1. PLO stands for pressure limiting orifice and AN stands for acceleration nozzle. All dimensions are in millimeters.

	**PLO**	**Lens 1**	**Lens 2**	**Lens 3**	**Lens 4**	**Lens 5**	**AN**
$D_{orifice}$	0.3	3.45	3.25	3.02	2.72	1.97	1.9
L_{spacer}	150	20	21	22	23	24	-

2.2 Simulations

In order to compute the particle trajectories, the flow field was first computed using the commercial CFD finite-element solver of COMSOL. The flow was assumed to be laminar, stationary, compressible, viscous and axisymmetric. The boundary conditions specified in Supplementary Figure 4 were used.

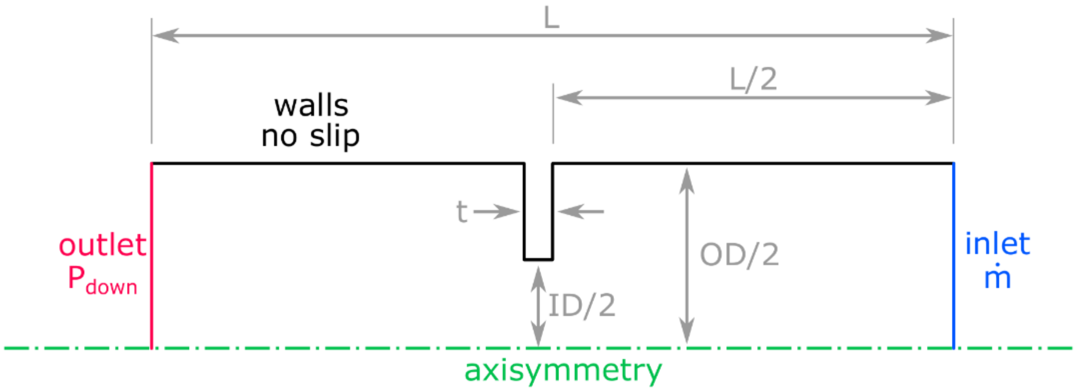

Supplementary Figure 4. Computational domain and boundary conditions to compute the velocity, density and pressure fields across an aerodynamic lens. $\dot{\boldsymbol{m}}$ is the mass flowrate and $\boldsymbol{P_{down}}$ is the downstream pressure.

The velocity, density and pressure fields are used to compute particle trajectory. The forces applied to the particles are the drag force $\vec{F}_d$ and the Langevin force $\vec{F}_L$.

$$m\vec{a} = \vec{F}_d + \vec{F}_L \tag{S1}$$

Where the drag force is given by:

$$\vec{F}_d = \frac{3\pi\mu_f d_p}{C_c}\vec{v}_r \tag{S2}$$

μ_f being the fluid dynamic viscosity, d_p the particle diameter, C_c the Cunningham correction slip factor and $\vec{v}_r$ the relative velocity.

The Langevin force is given by:

$$\vec{F}_L = \vec{\zeta}\sqrt{\frac{216\nu_f k_B T_f}{\pi\rho_f d_p^5\left(\frac{\rho_p}{\rho_f}\right)^2 C_c\Delta t}} \tag{S3}$$

Where $\vec{\zeta}$ is a zero-mean, unit-variance-independent Gaussian random vector, ν_f is the kinematic viscosity, k_B is the Boltzmann constant, T_f is the fluid temperature, ρ_f is the fluid density, ρ_p is the particle density and Δt is the time step.

This differential equation was derived using the velocity Verlet algorithm, and implemented in Python code. The numerical scheme of the lagrangian tracking has been presented elsewhere (Reynaud et al., 2021). In order to check this method, it has been applied to a specific lens simulated in the literature by (Wang et al., 2005) and showed good agreement (cf. Supplementary Figure 5 and Supplementary Figure 6).

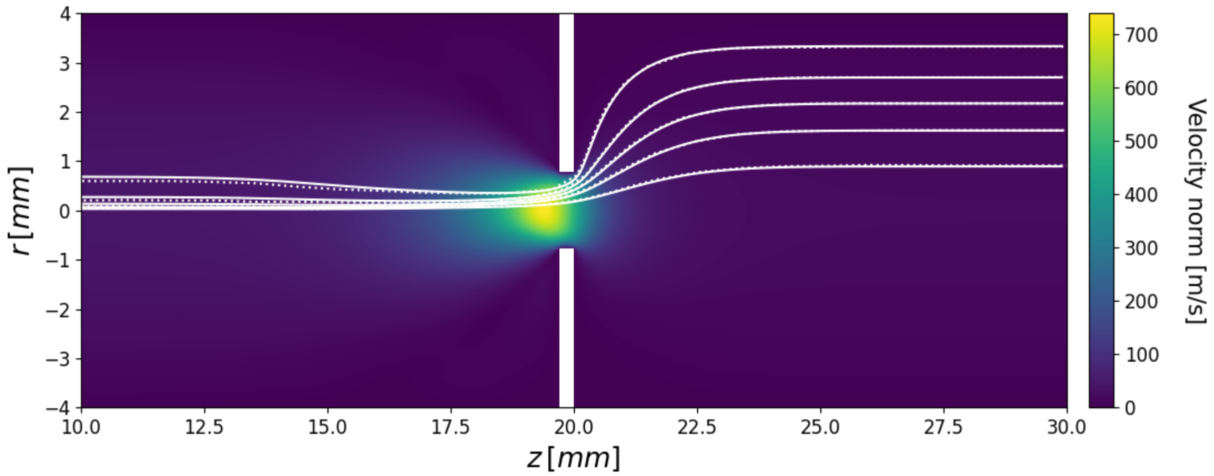

Supplementary Figure 5. Comparison between the trajectories computed by (Wang et al., 2005) (dashed lines) and this work (solid lines). The gas flows from the right to the left.

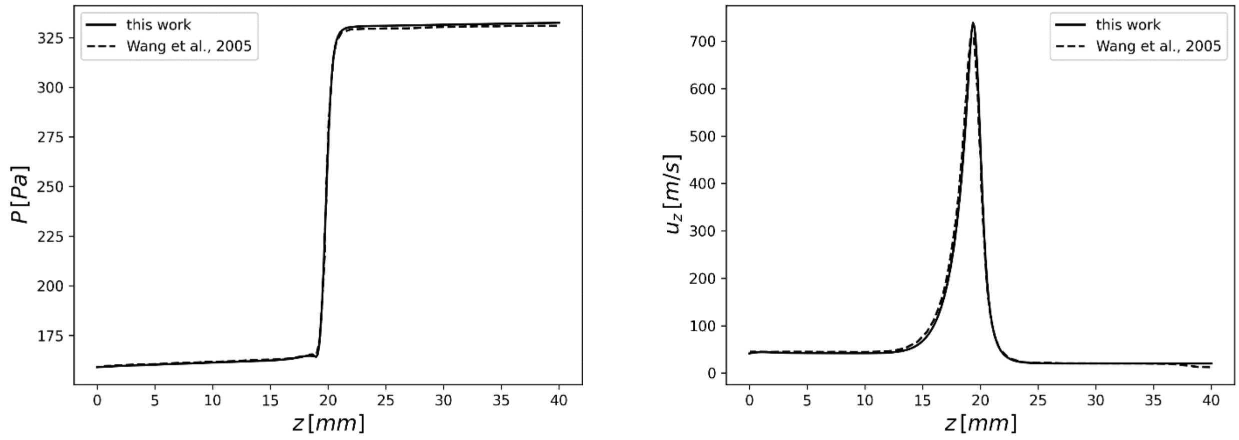

(**a**) (**b**)

Supplementary Figure 6. Comparison of the pressure (**a**) and axial velocity along the lens axis (**b**) between results obtained by (Wang et al., 2005) and this work.

To simulate the lens presented in this study, we used a typical mass flow rate of $\dot{m} = 3.8 \times 10^{-7}\ kg/m^3$.

3 Vacuum system modelling

The calculation of the pressures relies on the conservation of the throughput Q along the pumping stages. The system hereunder describes the throughput conservation between different points.

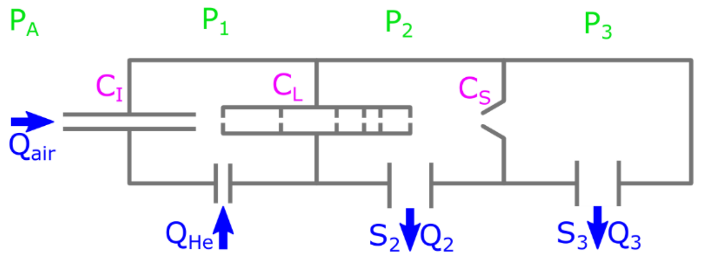

Supplementary Figure 7. Sketch of the vacuum system where $\boldsymbol{P_A}$ is the atmospheric pressure and $\boldsymbol{P_i}$ the pressure of the i-th chamber. $\boldsymbol{C_I}$, $\boldsymbol{C_L}$ and $\boldsymbol{C_S}$ are the conductances of the inlet, the aerodynamic lens and the skimmer, respectively. $\boldsymbol{S_2}$ and $\boldsymbol{S_3}$ are the turbomolecular pump speed, pumping down chamber 2 and 3, respectively.

$$\begin{cases} Q_{1\to 2} = Q_{2\to pump2} + Q_{3\to pump3} \\ Q_{A\to 1} + Q_{He} = Q_{1\to 2} \\ Q_{2\to 3} = Q_{3\to pump3} \end{cases} \quad \text{(S4)}$$

Where $Q_{1\to 2}$ is the throughput between chamber 1 at pressure P_1 and chamber 2 at pressure P_2. The equation system (S1) represents the vacuum system shown in Supplementary Figure 7, which includes two pumping stage and a helium throughput. This system has been simplified to model various scenarios. Using the relationships between throughput, pressures, pumping speeds and conductances:

$$\begin{cases} (P_1 - P_2)C_L = P_2 S_2 + P_3 S_3 \\ (P_A - P_1)C_I + Q_{He} = (P_1 - P_2)C_L \\ (P_2 - P_3)C_S = P_3 S_3 \end{cases} \quad \text{(S5)}$$

Where the inlet conductance in the viscous regime is given by:

$$C_I = \frac{\pi D_I^4}{128\mu_f L_I}\bar{p} \quad \text{(S6)}$$

Where D_I and L_I are the inner diameter and the length of the inlet capillary, μ_f is the dynamic viscosity, which is a function of the temperature given by Sutherland's law and $\bar{p} = \frac{P_A+P_1}{2}$. With the appropriate values, $C_I = 2.85 \times 10^{-3}\ l/s$.

The lens conductance is given by:

$$\frac{1}{C_L} = \sum_i \frac{1}{C_{orifice,i}} + \frac{1}{C_{spacer,i}} \quad \text{(S7)}$$

Where $C_{orifice,i} = 20\pi r_{orifice,i}^2$ and $C_{spacer,i} = 12.1 d_{spacer,i}^3 / L_{spacer,i}$. It leads to the value $C_L = 1.26 \times 10^{-2}\ l/s$.

And the skimmer conductance is $C_S = 11.6A$, A being the area of aperture. Thus, $C_S = 2.32\ l/s$.

Supplementary Table 4. Comparison between the model and the measured pressures. The turbo pumping speed is $\boldsymbol{S_3 = 45}$ l/s, the primary pumping speed is $\boldsymbol{S_2 = 0.7}$ l/s and the inlet capillary is 11 cm long, heated at 85 °C.

Chamber	Model<br>[Torr]	Measured<br>[Torr]
**P1**	131	151
**P2**	0.90	2.2
**P3**	0.023	0.0087

4 Prototype

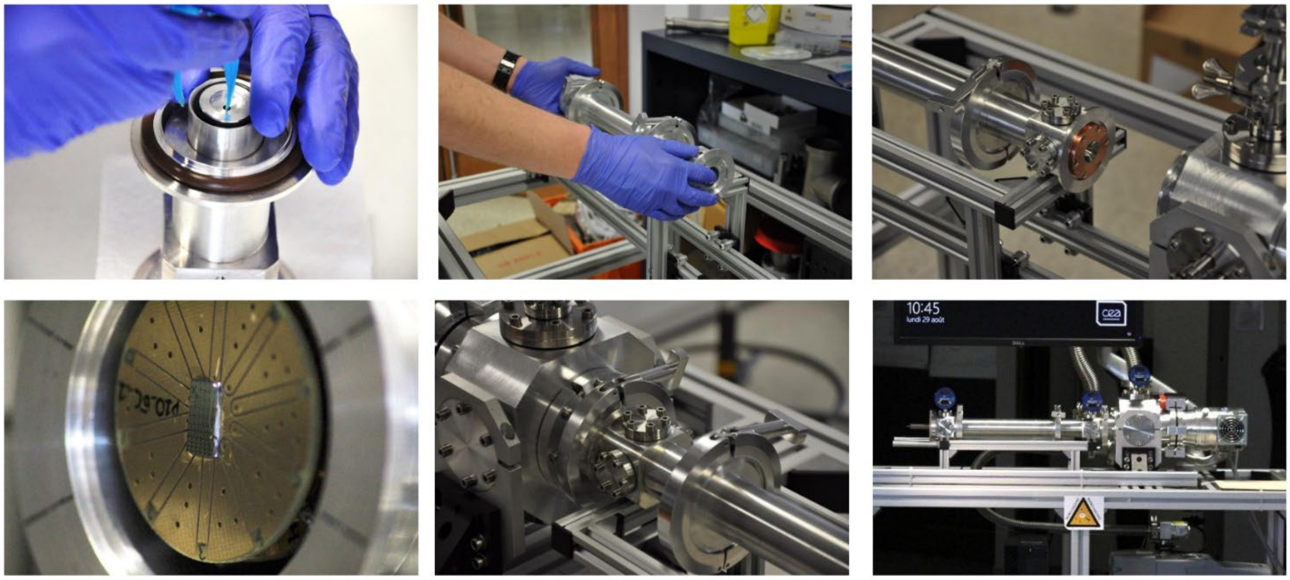

Supplementary Figure 8. Photographs of the prototype mechanical assembly.

5 Mass detection

The raw data were filtered in terms of:

- Mass: anomalous mass values below 2 MDa and above 1000 MDa were discarded
- Resolution: detection events with resolution over 5 times the noise standard deviation were discarded
- Position: events localized to be in the range 0.24 L_{NEMS} and 0.48 L_{NEMS} were selected

- Desorption events: frequency jumps towards higher frequency were discarded

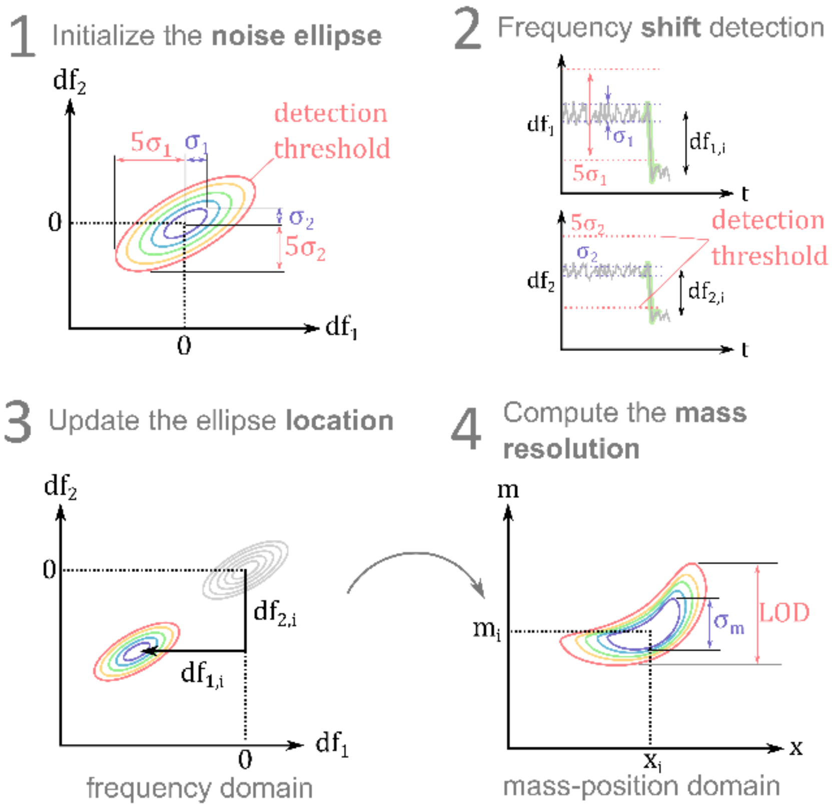

Supplementary Figure 9. Illustration of the frequency jump to mass and position calculation process. (1) The noise is characterized by evaluating $\boldsymbol{\sigma_1}$ and $\boldsymbol{\sigma_2}$, leading to the noise ellipse. (2) The whole frequency trace is swept to detect the shifts corresponding to landing events and the mass resolution is computed by (3) updating the resonance frequency and (4) extracting the extrema values of the noise ellipsoid in the mass-position domain.

6 Energy dissipation in nano-resonators

Using the model developed by (Trzpil et al., 2021), the contribution of each phenomena over the quality factor were quantified. The calculations were done for a 9 µm long, 300 nm wide and 160 nm thick beam. The lower value is the one due to the support and compares well with the measurement under vacuum. Those results indicate that the energy losses through the support are predominant over thermoelastic and acoustic losses.

**NEMS #1**<br>length = 9 µm	
$f_{0,1}$	28.4 MHz
$Q_{support}$	4.65×10^3
$Q_{thermoelastic}$	2.12×10^5
$Q_{acoustic}$	1.13×10^{13}

7 Polystyrene deposition and image analysis

Polystyrene nanoparticle solution (batch PS3338A-0416, Magsphere, USA) was nebulized and analyzed using an SMPS (scanning mobility particle sizer, model 3380, TSI). The resulting granulometry is shown on Supplementary Figure 10. To estimate the location of the peaks, a gaussian fit has been used. Two peaks can be observed: 19.7 nm and 90.2 nm. The first peak was attributed to salt clusters from the solvent, as it was present even in the absence of nanoparticles.

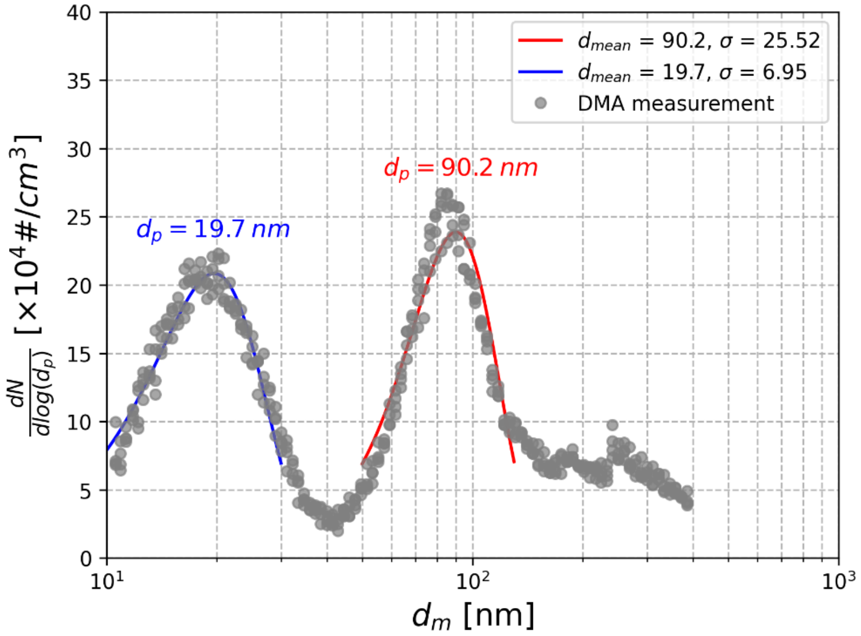

Supplementary Figure 10. Granulometry of the 100 nm polystyrene nanoparticle measured using an SMPS.

The deposit was analyzed using image processing of the photograph taken through a binocular. It was then converted to grey scale, and the pixel size was measured in millimeter thanks to a reference scale simultaneously imaged (every tick is 0.1 mm). Profiles were then plotted along the x and y axis. Since the particle density was relatively low, the profile results in discrete high value peaks, yielding slightly noisy profiles. A moving average was applied to smooth the gaussian-like curves and facilitate the FWHM measurement (300 µm).

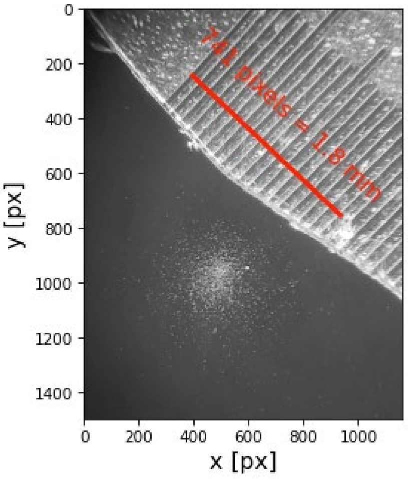

(**a**)

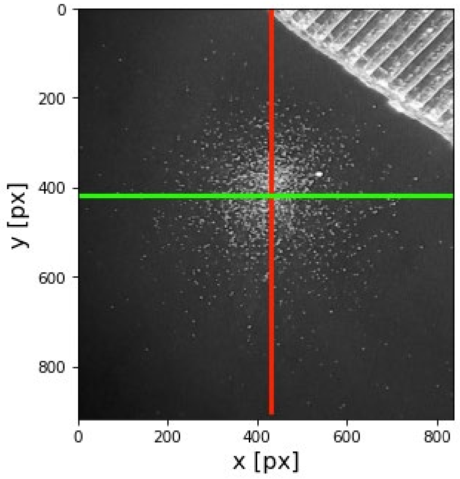

(**b**)

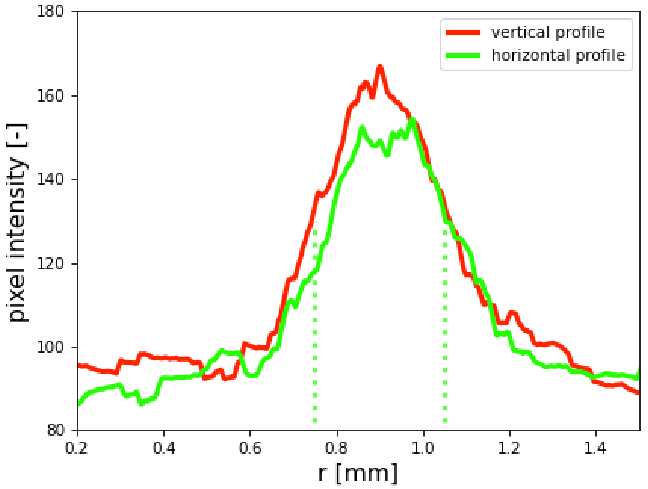

(**c**)

Supplementary Figure 11. Process to measure the size of the particle deposit. (**a**) Deposit pattern and scale used to the calibration. (**b**) Cropped image and profile location. (**c**) Vertical and horizontal profiles.

8 Gold nanoparticle mass measurement with the new prototype

Gold colloidal samples were diluted by a factor 2 in methanol to facilitate nESI nebulization process. In order to compute the particle capture efficiency, the number of sprayed particles was derived from the concentration C, the flow rate Q and the duration of the NEMS exposure Δt. The ratio between the number of sprayed particles and the number of detected particle ratio was then computed for individual NEMS devices using the relationship:

$$capture\ efficiency = C \times Q \times \Delta t / N_{detected} / N_{NEMS} \quad \text{(S8)}$$

Details of gold nanoparticle mass measurement experiments are reported in **Supplementary Table 3**. In order to quantify the improvement factor between the first-generation prototype and this work, the

experiments of (Dominguez-Medina et al., 2018) were used. We only took into account the experiments featuring ESI nebulization, since this is the technique that has been used to spray GNP. The capture efficiency was computed for one single NEMS since the number of used devices can vary from an experiment to the other and the objective was to compare performances of NEMS-MS system generations.

Supplementary Table 5. Experimental parameter and capture efficiency computed for the gold nanoparticles. [1]Range computed based on the upper and lower capture efficiency reported by (Dominguez-Medina et al., 2018), namely 1 per $\sim\mathbf{2}\times\mathbf{10^9}$ and 1 per $\sim\mathbf{1}\times\mathbf{10^{10}}$ sprayed particle, respectively.

GNP sample	Sprayed concentration	Flow rate	ESI voltage	Duration	Sprayed particles	Number of NEMS	Event count	Capture efficiency	Capture efficiency for 1 NEMS	Improvement factor[1]
	[$\times 10^{10}$ NP/ml]	[µl/min]	[kV]	[s]	[$\times 10^9$ NP]	[-]	[-]	[-]	[-]	[-]
**20 nm** (BBI)	35	0.5	2.3	4200	12.3	9	1220	1 per 4.5×10^6	1 per 9.0×10^7	22 – 111
**30 nm** (Sigma-Aldrich)	8.95	0.55	2.5	3800	3.12	12	739	1 per 2.5×10^6	1 per 5.1×10^7	40 – 198
**40 nm** (BBI)	4.50	0.5	2.2	4200	1.58	9	183	1 per 3.9×10^6	1 per 7.8×10^7	26 – 129

References

Dominguez-Medina, S., Fostner, S., Defoort, M., Sansa, M., Stark, A.-K., Halim, M.A., Vernhes, E., Gely, M., Jourdan, G., Alava, T., Boulanger, P., Masselon, C., Hentz, S., 2018. Neutral mass spectrometry of virus capsids above 100 megadaltons with nanomechanical resonators. Science 362, 918–922. https://doi.org/10.1126/science.aat6457

Reynaud, A., Leblanc, M., Ranarivelo, V.B., Zinola, S., Breuil, P., Viricelle, J.P., 2021. How does the dielectrophoresis affect the soot dendrite growth on resistive sensors? Sens. Actuators Phys. 327, 112729. https://doi.org/10.1016/j.sna.2021.112729

Trzpil, W., Maurin, N., Rousseau, R., Ayache, D., Vicet, A., Bahriz, M., 2021. Analytic Optimization of Cantilevers for Photoacoustic Gas Sensor with Capacitive Transduction. Sensors 21, 1489. https://doi.org/10.3390/s21041489

Wang, X., Gidwani, A., Girshick, S.L., McMurry, P.H., 2005. Aerodynamic Focusing of Nanoparticles: II. Numerical Simulation of Particle Motion Through Aerodynamic Lenses. Aerosol Sci. Technol. 39, 624–636. https://doi.org/10.1080/02786820500181950
